# Treg activation defect in type 1 diabetes: correction with TNFR2 agonism

**DOI:** 10.1038/cti.2015.43

**Published:** 2016-01-08

**Authors:** Yoshiaki Okubo, Heather Torrey, John Butterworth, Hui Zheng, Denise L Faustman

**Affiliations:** 1 Immunobiology Department, Massachusetts General Hospital, Harvard Medical School, Boston, MA, USA; 2 Department of Biostatistics, Massachusetts General Hospital, Boston, MA, USA

## Abstract

Activated T-regulatory cells (aTregs) prevent or halt various forms of autoimmunity. We show that type 1 diabetics (T1D) have a Treg activation defect through an increase in resting Tregs (rTregs, CD4^+^CD25^+^Foxp3^+^CD45RA) and decrease in aTregs (CD4^+^CD25^+^Foxp3^+^CD45RO) (*n*= 55 T1D, *n*=45 controls, *P*=0.01). The activation defect persists life long in T1D subjects (T1D=45, controls=45, *P*=0.01, *P*=0.04). Lower numbers of aTregs had clinical significance because they were associated with a trend for less residual C-peptide secretion from the pancreas (*P*=0.08), and poorer HbA1C control (*P*=0.03). In humans, the tumor necrosis factor receptor 2 (TNFR2) is obligatory for Treg induction, maintenance and expansion of aTregs. TNFR2 agonism is a method for stimulating Treg conversion from resting to activated. Using two separate *in vitro* expansion protocols, TNFR2 agonism corrected the T1D activation defect by triggering conversion of rTregs into aTregs (*n*=54 T1D, *P*<0.001). TNFR2 agonism was superior to standard protocols and TNF in proliferating Tregs. In T1D, TNFR2 agonist-expanded Tregs were homogeneous and functionally potent by virtue of suppressing autologous cytotoxic T cells in a dose-dependent manner comparable to controls. Targeting the TNFR2 receptor for Treg expansion *in vitro* demonstrates a means to correct the activation defect in T1D.

Type 1 diabetes (T1D) is an autoimmune disease characterized by destruction of the insulin-secreting islets of Langerhans, resulting in hyperglycemia. Although the destruction of insulin-secreting islets is carried out by antigen-specific cytotoxic CD8 T cells, CD4 T regulatory cells (Tregs) are important for disease prevention. Abundant evidence shows that higher numbers of Tregs prevent or halt many diverse and spontaneous forms of autoimmunity, especially in animal models.^1, 2^ Tregs are commonly identified as CD4^+^CD25^+^Foxp3^+^ cells, but within peripheral T cells there are two functionally heterogeneous subgroups defined by expression of CD45RA or RO.^3, 4^ One subgroup is resting Tregs (rTregs, CD4^+^CD25^+^Foxp3^+^CD45RO^−^RA^+^) and the other is activated Tregs (aTregs, CD4^+^CD25^+^Foxp3^+^CD45RA^-^RO^+^).^5^ When rTregs are converted to aTregs, they display high expression of tumor necrosis factor (TNF) receptor 2. aTregs expressing high TFNR2 constitute the most immunologically suppressive Treg subgroup.^6, 7, 8, 9, 10, 11^

The TNFR2 receptor is a signaling protein that triggers Treg differentiation from resting to activated state and triggers Treg proliferation in normal mice and humans.^8, 9^ *In vitro* application of TNF or TNFR2 agonistic antibodies produces potent and homogeneous aTregs with high TNFR2 expression.^6, 8^ In functional assays, aTregs with high expression of TNFR2 are highly immunosuppressive, which makes them desirable for autoimmunity but not for cancer treatment. An overabundance of aTregs expressing TNFR2 drives cancer in mice and humans.^11, 12, 13, 14^

Previous research shows alterations in Tregs numbers or function in human autoimmunity. Tregs have a central role in the maintenance of the immune balance to prevent autoimmunity. Direct mutations in Foxp3 gene culminate in severe immune deregulation and polyglandular forms of autoimmunity called the human IPEX syndrome or in the mouse, the Scurfy mouse.^15, 16^ In lupus, there is an activation defect yielding an overabundance of rTregs and an underabundance of aTregs.^5^ In type 1 diabetes (T1D), early studies reported reductions in Treg absolute numbers, but more recent, larger and methodologically driven studies have not found such alterations. The more recent studies used new Treg markers such as CD127^−^ that more finely distinguish Treg populations and examined Tregs in all age groups with age-matched controls.^17, 18^ Recent studies of T1D suggest that Treg function might be altered. Despite normal numbers of Tregs in T1D, the Tregs were found to be more susceptible to apoptosis by low IL2 concentration. IL2 is obligatory for Treg survival.^18^ A recent study found a functional disturbance in aTregs in T1D.^19^ This suggests that more refined phenotyping and functional studies of human Tregs in T1D are needed to understand their possible pathogenic role in autoimmunity.

As early as 1989 it was reported that T1D T lymphocytes (T cells) have developmental or differentiation defects related to the CD45 protein and its splice variants, CD45RA and CD45R0.^20^ Follow-up studies confirm this finding in diabetes.^21^

This defect in an over abundance of resting T cells defined with CD45RA (formerly 2H4^+^) was not restricted to T1D but extends broadly in autoimmune diseases such as autoimmune atopic dermatitis and inflammatory bowel disease.^22, 23^ CD45RA resting T cells were originally called T4^+^2H4^+^ cells (naïve cells) and CD45RO activated cells were originally called T4^+^4B4 cells (memory cells) before the standardization with CD markers. When examining all peripheral T cells, T1D subjects have an abundance of resting CD45RA T cells with poor *in vivo* conversion to activated CD45RO expressing T lymphocytes.^20, 24, 25^ Decreased numbers of activated CD45RO T cells and increased numbers of resting CD45RA T cells are present in established T1D and also in prediabetic subjects.^24, 26, 27^ This finding is highly reproducible^28, 29^ and has been extended to young children with pre-diabetes defined as low C-peptide and at high risk for future progression to hyperglycemia (but still normoglycemic).^30^

This study addresses three major questions. First, does T1D display a primary defect preventing activation of rTregs to aTregs? Second, if there is a defect, is it clinically significant? Third, can this defect be corrected with TNFR2 agonism? TNFR2 is the primary receptor for Treg differentiation *in vivo* from resting to activated state.^8, 9, 31^

## Results

### T1D subjects have lower numbers of aTregs and higher numbers of rTregs

**Figure 1:**
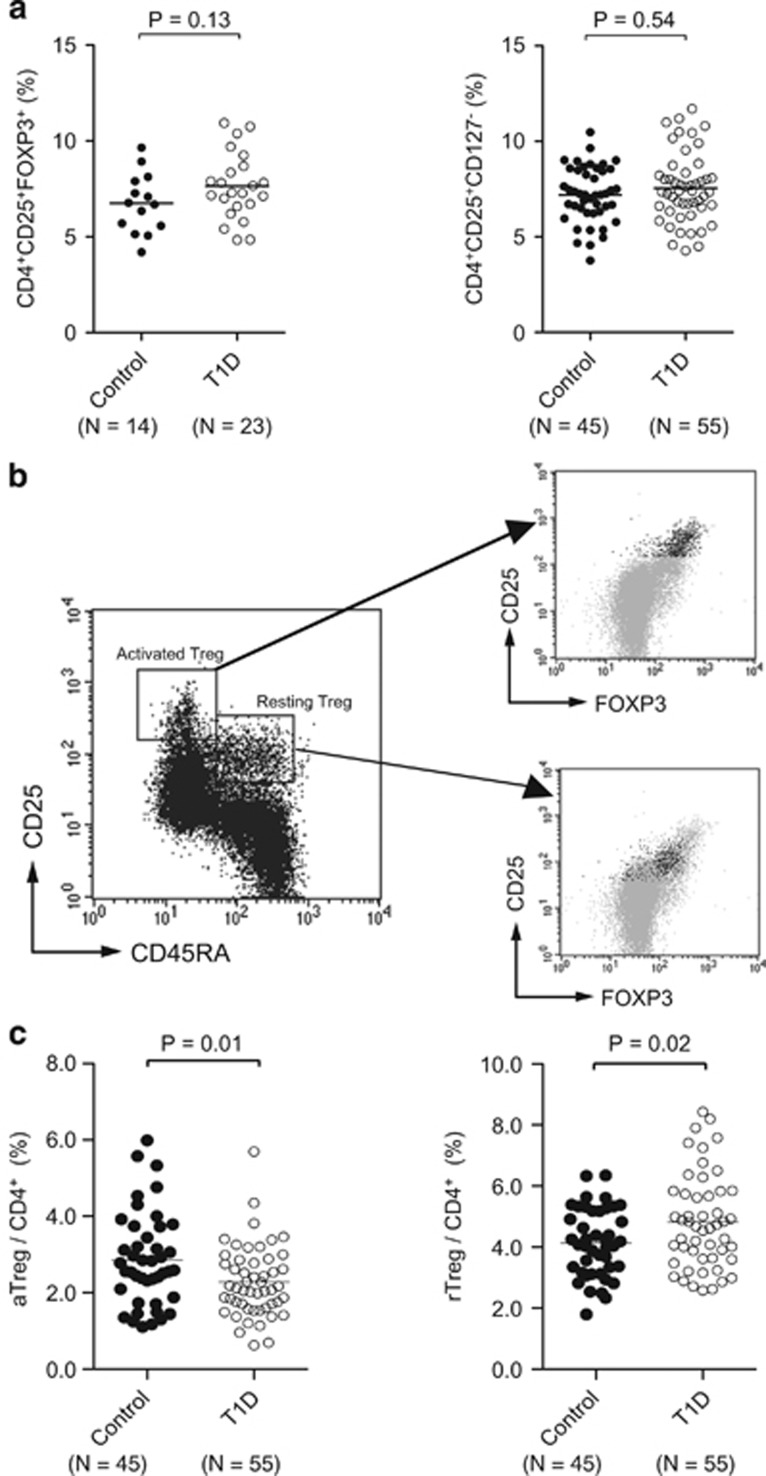
The frequency of Treg cells in T1D compared with controls. (**a**) The data shows the characterization of human Tregs in type 1 diabetic subjects (*n*=14) compared with control subjects (*n*=23). The frequency of Tregs is quantified by the percentage of CD4^+^CD25^+^Foxp3 (*P*=0.13) or quantified by the percentage of CD4^+^CD25^+^CD127^−^ (*P*=0.54) of controls compared with T1D (*n*=45 controls: *n*=55 T1D). (**b**) Flow cytometric analysis gating methods to quantify activated and resting Tregs by staining with CD45RA antibodies. The gating method shows the existence of a subpopulation of resting Tregs (rTregs) with high CD45RA^+^, or a subpopulation of activated Tregs (aTregs) that are CD45RA^−^. The gating also shows that the CD25 cell surface marker has high expression in aTreg and a lower expression in rTregs. These gates were applied to CD4^+^CD25^+^CD127^−^ Tregs and allowed the quantification of aTregs versus rTregs. The rTregs also have slightly lower levels of Foxp3. (**c**) The characterization of activated and resting Tregs from T1D subjects (*n*=55) compared with control subjects (*n*=45). The statistics shows the trends of type 1 diabetics lower numbers of aTregs (*P*=0.01) and higher numbers of rTregs (*P*=0.02).

We first examined the frequency of Treg cells in the peripheral blood of T1D subjects compared with control subjects using standard flow cytometric methods. As previously reported, using conventional methods of measuring Tregs with CD4, CD25, Foxp3 and CD127 markers, we did not observe differences in the frequency of Tregs (CD4^+^CD25^+^FOXP3^+^ or CD4^+^CD25^+^CD127^−^ cells) between T1D and control subjects (Figure 1a, *n*=12 controls, *n*= 23 T1D, *P*=0.13, *P*=0.54). However, when CD4^+^CD25^+^ cells were divided into two subpopulations depending on expression of CD45RA, T1D subjects had significantly fewer aTregs and significantly more rTregs (Figures 1b and c). This was true whether the aTregs were quantified as a subpopulation within the total CD4 population (Figure 1c, left, *P*=0.01) or whether the rTregs were studied as a subpopulation within the total CD4 population (Figure 1c, right, *P*=0.02). The data represented as paired samples shows the reproducibility of the aTreg defect in T1D compared with controls (Supplementary Figure 1).

### Low numbers of aTregs in T1D persist over the life span

**Figure 2:**
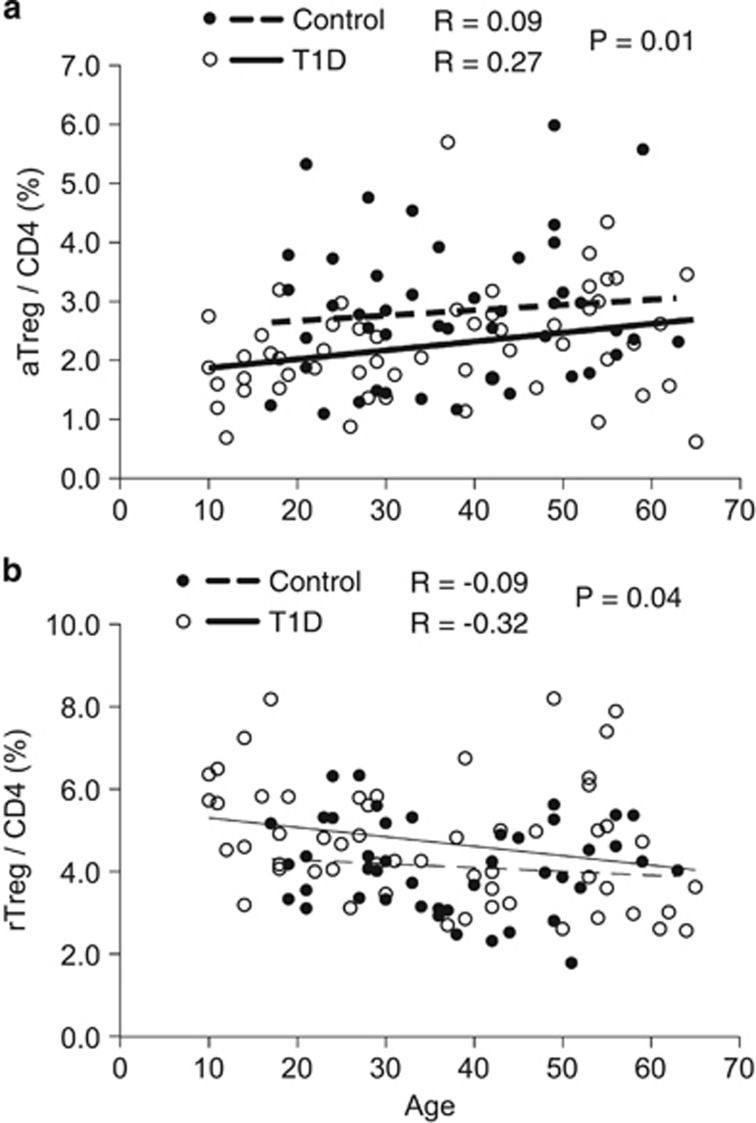
Quantification of rTregs and aTregs in T1D across the life span compared with controls. (**a**) In T1D subjects and controls, aTregs (CD4^+^CD25^+^Foxp3^+^CD45RO) were compared across the life span (*n*=55 type 1 diabetes; *n*=45 control subjects; *P*=0.01). (**b**) rTregs (CD4^+^Foxp3^+^CD25^+^CD45RA) across the life span were also compared with controls (*n*=55 type 1 diabetes; *n*=45 control subjects; *P*=0.04).

It is well-recognized that some protein markers on lymphocytes change with age. The CD45RA cell activation marker on T lymphocytes decreases over the life span, while the CD45RO activation marker increases over the life span.^32, 33^ Consequently, aTregs and rTreg cells were studied over the lifespan in control and T1D subjects. The lifespan for these studies was defined from donor subjects from 16 years and upward for the control group and from 8 and upward for the T1D group. Figure 2a shows that T1D patients exhibit a significant decrease in the proportion of aTregs (CD4^+^CD25^+^CD127^−^CD45RO)/CD4 cells over the life span, and their values over time are consistently lower than those in controls (*n*=55 T1D; *n*= 45 controls, *P*=0.01). Conversely, Figure 2b shows that TID patients exhibit a significant increase in the proportion of rTregs/CD4 cells over the life span, and their values over time are consistently higher than those in controls (*n*=55 T1D; *n*=45 controls; *P*=0.04). These *P-*values are the comparisons of the intercepts in the two linear trends. This is based on the assumption that the slopes of Tregs over aging in the control and T1D are the same. This assumption is supported by the test of the difference of slopes resulting in non-significant differences in either Figure 2a (*P*=0.69) or Figure 2b (*P*=0.47). The data reveal that the Treg activation defect in T1D persists regardless of age (Figures 2a and b). The data show that there is a significant difference in these measures between the control and T1D groups regardless of age. Two statistical tests using linear regression models support this conclusion. (1) A test of interaction between age and group showed there is no significant difference between the slopes of the trend lines in these groups. (2) A comparison of the intercepts in the two trend lines, assuming a common slope, gives the two *P*-values shown in Figure 2a, *P*=0.01, and in Figure 2b *P*=0.04. *P-*values were calculated by ANOVA and *R*-values were calculated by Spearman correlation.

### Low numbers of aTregs in T1D are associated with lower levels of residual insulin secretion and poorer glycemic control

**Figure 3:**
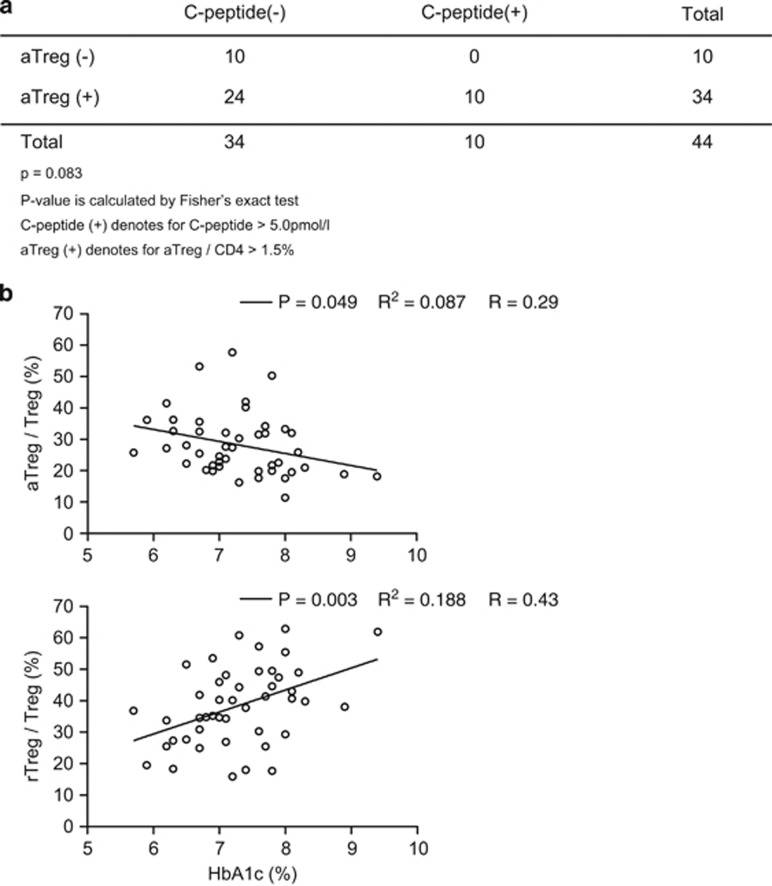
Clinical significance of low numbers of aTregs in type 1 diabetes as it relates to remaining C-peptide and HbA1c control. (**a**) Using the ultrasensitive assay of C-peptide to detect low levels in serum, T1D subjects were classified into presence or absence of C-peptide (presence is defined by >5.0 pmol l^−1^). Likewise T1D subjects were classified into presence (>1.5% aTregs/CD4) or absence (<1.5% aTregs/CD4) of aTregs (*P*=0.08). (**b**) In type 1 diabetes subjects, aTregs strongly correlated with lower HbA1c numbers, a correlate of blood sugar control (*P*=0.01, *n*=45). High numbers of rTregs correlated with high HbA1c values (*P*=0.004, *n*=45). The *R* values is 0.29 in **b** or *R*^2^ of 0.087 for the upper plot. The *R* value is 0.43 in **b** or *R*^2^ of 0.188 for the lower plot.

We studied the clinical significance of low numbers of aTregs by comparing T cell abundance with two different measures of islet function, C-peptide secretion and HbA1c control. C-peptide, the co-peptide secreted with insulin that is a marker for endogenous insulin secretion, can be measured in ultrasensitive serum assays, with detection levels as low as 5 pmol l^−1^.^34, 35^ We first examined C-peptide by dividing 44 T1D subjects by presence or absence of C-peptide and looked at the association with the presence or absence of aTregs (absence of aTregs was defined as aTregs/CD4 cells <1.5%, while presence of aTregs was defined as >1.5%.). Figure 3a shows a trend, although not statistically significant, toward presence of aTregs with presence of C-peptide (*P*=0.08). In contrast, absence of aTregs was associated with no residual C-peptide levels (*P*<0.05). These data support, but do not prove, the hypothesis that aTregs are protective or at least are associated with more residual pancreatic islet activity.

We next examined the frequency of aTregs in relation to glycemic control. Glycemic control is most commonly tracked by measuring HbA1c levels, with low HbA1c levels reflecting better control. Previous research found that even low levels of C-peptide with new ultrasensitive C-peptide assays are protective of glucose excursions and highly protective of improved HbA1c control.^34^ The data in Figure 3b show that higher levels of aTregs were associated with lower HbA1c values (*P*=0.049, *n*=45). Conversely, higher levels of rTregs were associated with poorer HbA1c control (*P*=0.003, *n*=45). The *R* values is 0.29 in Figure 3b or *R*^2^ of 0.087 for the upper plot. The *R* value is 0.43 in Figure 3b or *R*^2^ of 0.188 for the lower plot.

The data support the concept that having more aTregs is desirable in T1D and that the activation defect might have a clinical significance.

### Agonism through TNFR2 expands T1D Tregs to potent aTregs

**Figure 4:**
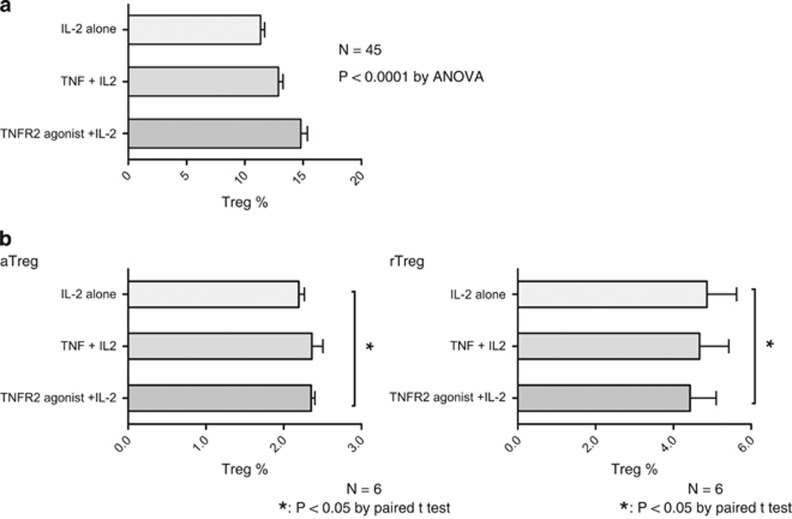
Short-term culture (48 h) of human diabetic and control CD4^+^ T cells cultured with IL2 alone, TNF plus IL2 or with TNFR2 agonist plus IL2 expand into aTregs, an evaluation of Treg percentages. (**a**) In freshly isolated CD4^+^ cells from human T1D and controls, CD4^+^CD25hiCD127^−^ Treg expand, and most vigorous expansion is observed with TNFR2 agonism in a 48-h protocol (*n*=30 type 1 diabetics, *P*<0.000, *n*=15 controls, *P*<0.000). (**b**) T1D-expanded Tregs are preferentially aTregs, not rTregs, with all three expansion conditions (*n*=6, *P*<0.05).

The presence of the TNFR2 receptor on Tregs identifies a subgroup of the most potent Tregs with maximal immunosuppressive function.^7, 8, 9, 10^ TNFR2 agonism, if combined with IL2, acts as a master switch to expand Tregs *in vitro* in normal volunteers.^8^ We sought to extend this observation to TID patients using a short-term culture assay. We cultured short term T1D CD4 T cells for 48 h with IL2 alone, TNF plus IL2, and TNFR2 agonistic antibody and IL2. Both IL2 and TNF are known agonists for Treg expansion but the addition of TNFR2 agonistic antibodies in normal subjects results in selective expansion of the most potent Tregs.^8^ In T1D the percentage of Tregs, defined as CD4^+^Foxp3^+^CD127^−^ cells, increased with 48 h of culture from a baseline with IL2 alone of 11.6±0.4% to a baseline of Tregs with TNF and IL2 to 13.6±0.37% and to a Treg-expanded population of 16.3±0.61% with a TNFR2 agonist with IL2. The data was statistically significant for successful TNFR2 Treg expansion in T1D compared with IL2 alone (*n*=45 samples, *P*<0.001, *P*<0.001; Figure 4a). Also as a control, non-diabetic subjects were also studies in the Treg short-term expansion assays in the same three culture conditions. Similar to the T1D, control subject cells expanded with IL2 and the additional of TNFR2 agonist allowed even greater Treg expansion (Figure 4a; *n*=15, *P*=0.015).

We next examined in the same short-term 48 h assay which subgroups of Tregs—aTregs or rTregs—were responsible for the increase in overall Tregs numbers. Greater numbers of aTregs were found with TNFR2 agonist plus IL2, versus IL2 alone or TNF plus IL2, while fewer numbers of rTregs were found (*n*=6, *P*<0.05; Figure 4b). Therefore T1D Tregs were responsive to the TNFR2 agonism and expansion of aTregs was preferentially observed (Figure 4b). In these short-term culture experiments, both TNF plus IL2 or TNFR2 agonism with IL2 were equally effective at aTreg appearance although overall Tregs numbers were greater with the TNFR2 agonist.

### T1D Tregs expand with TNFR2 agonism and become aTregs

**Figure 5:**
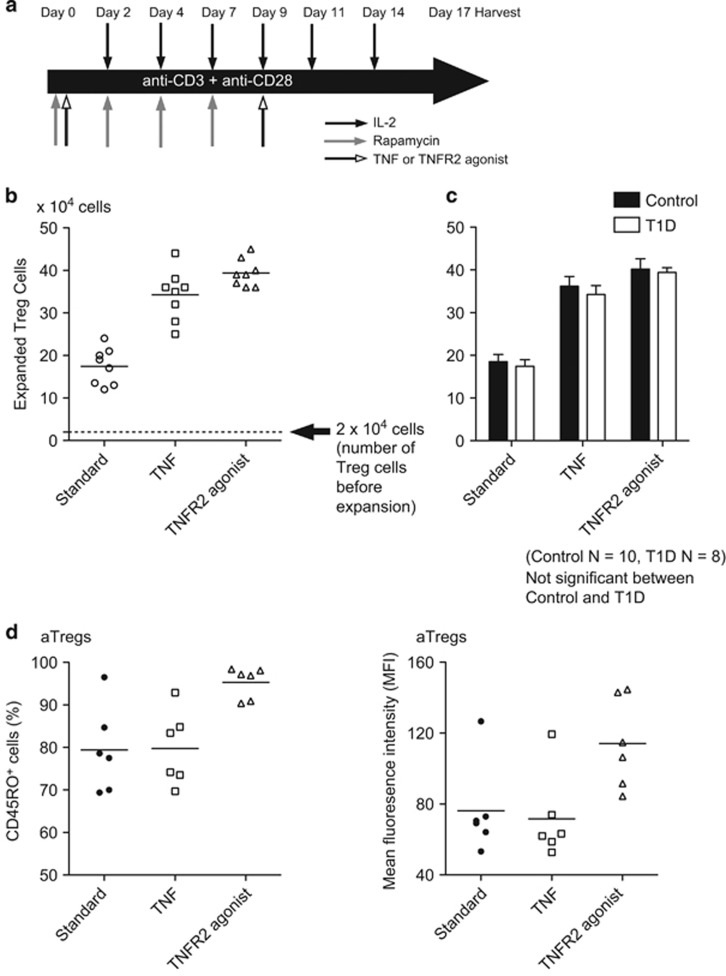
Extended Treg expansion protocol (17 days) to observe the effects TNFR2 agonism on vigorous Treg expansion and preferential expansion of aTregs on T cells from established type 1 diabetic subjects. (**a**) Protocol for purifying Tregs from fresh blood and expansion for 17 days. (**b**) Cell counts of purified Tregs from T1D patients, by treatment group, shows the effect of TNFR2 agonism induced Tregs (*n*=8, *P*<0.0001, standard expansion compared with TNFR2 agonist expansion using ANOVA with repeated measurements). (**c**) Data from T1D Tregs (*n*=10) and control Tregs (*n*=8) expansion with the standard protocol, with TNF and with TNFR2 agonism (*n*= 10 controls, *n*=8 type 1 diabetics, *P*<0.05). T1D Treg cells expanded as vigorously as control T cells using ANOVA with repeated measurements, *P*-value for interaction=0.89. (**d**) T1D Treg expansion with TNFR2 agonism and impact on the numbers of aTregs as defined with a large percentage of CD45RO^+^ cells and with a high mean fluorescence intensity of aTregs as CD45RO (*n*=6, *P*<0.001) using a paired *t-*test. Data studied by the ANOVA with repeat measurements using random effects model with Dunnett-Hsu *post hoc* showed testing the aTregs (left) had a *P*-value of 0.21; the rTregs had a *P*-value of 0.003. All diabetic samples are from long term type 1 diabetics.

We examined in more detail the effect of TNFR2 agonism on expansion of T1D Treg cells and differentiation into aTregs with more long-term culture. As mentioned previously, all type 1 diabetics used in these studies had disease duration greater than 2 years after the diagnosis. We positively selected CD4^+^CD25^+^ cells using magnetic beads, and expanded the cells with anti-CD3 and anti-CD28 mAb bound magnetic beads in presence of IL-2, rapamycin (the standard expansion), TNF plus IL-2 or TNFR2 agonistic mAbs plus IL-2 for (Figure 5a). This was a longer expansion period of 17 days (Figure 5a). T1D Tregs had increased cell numbers of Treg with the greatest expansion with long-term culture with the addition of TNFR2 agonistic antibodies (Figure 5b). Moreover, Tregs expanded with TNFR2 agonist had greater cell numbers than expansion with standard protocol or TNF (Figure 5b, *n*=8, *P*<0.001, in each case using ANOVA with repeated measurements). Control CD4^+^CD25^+^ T cells from control subjects were also subjected to the same extended standard and TNF and TNFR2 agonist supplemental expansion protocol to determine whether there was any difference between T1D and controls. The data shown in Figure 5c also show that T1D Treg cells expanded as vigorously as control T cells (*n*=10 control, *n*=8 T1D, *P*-value for comparison=0.69 using ANOVA with repeated measurements, *P*-value for interaction=0.89).

Next we examined the phenotypes of expanded T1D Tregs during the 17 days of culture. Nearly 100% of expanded Tregs in every group exhibited Treg signature markers, such as CD25, FOXP3 and CTLA4 (data not shown). T1D cells expanded with TNFR2 agonism had higher levels of CD45RO^+^ cells compared with the standard expansion group, as measured by cell number counts or mean fluorescence intensity (Figure 5d; Supplementary Figure 2; *n*=6, *P*<0.001). The TNFR2 agonism uniquely promotes differentiation of Treg cells into the most activated state of CD45RO expressing cells, aTregs and this can be observed at day 17 of the cell collection. Also TNFR2 agonism in T1D is unique in expansion to create homogenous populations of these desired Treg cells unlike the standard protocols of anti-CD3 plus anti-CD28, IL2 and rapamycin that expand but as demonstrated in Figure 5d, expand heterogenous CD4 cells populations, not only highly activated Tregs.

### TNFR2-agonist treated Tregs from T1D are potent CD8 T-cell suppressors

**Figure 6:**
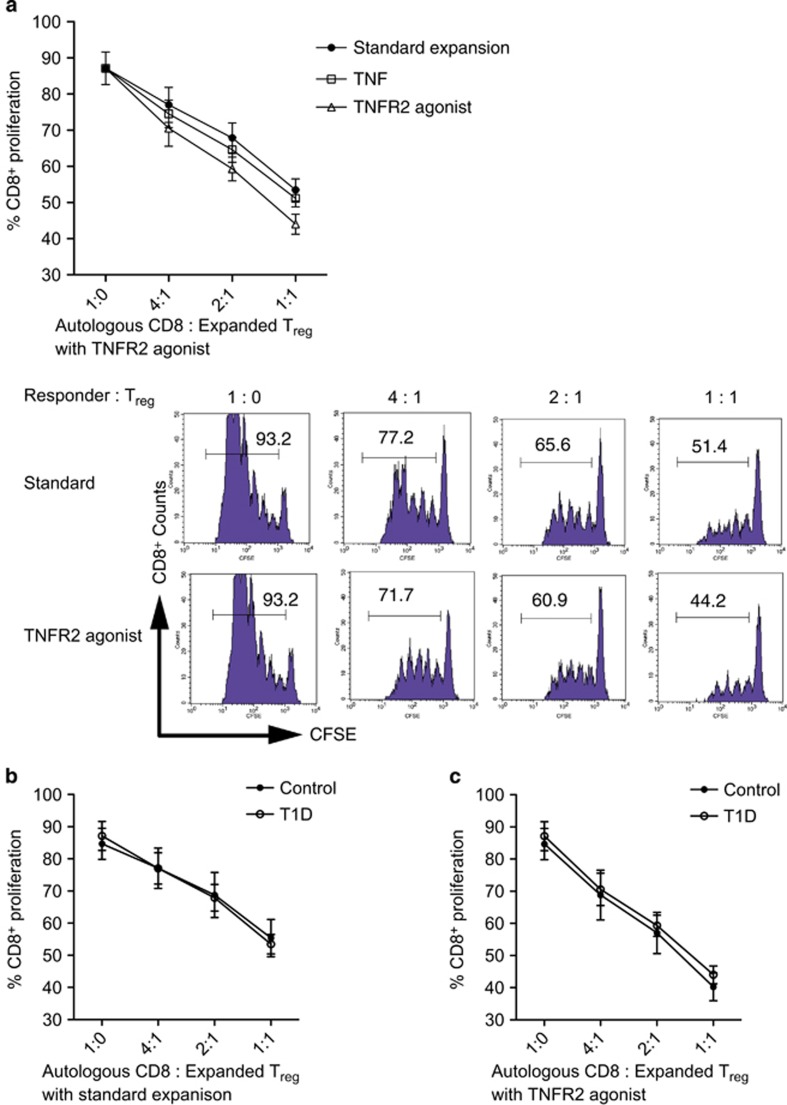
Functional assay of expanded T1D and control Tregs on autologous CD8 T-cell proliferation. (**a**) T1D Tregs expanded with the standard expansion, with standard expansion plus TNF or plus TNFR2 agonist in a dose-response manner to access inhibition of autologous CD8^+^ cells stimulated with anti-CD3 and IL-2 (*N*=5, *P*<0.0001). In a representative case, TNFR2 agonist treated Tregs dose-dependent suppression of CD8^+^ cell numbers is presented (bottom histograms) (**b**, **c**). T1D and control Tregs expanded with standard expansion (**b**) illustrate suppress of self (*n*=5 T1D and controls, *P*>0.05), T1D, and control Tregs expanded with TNFR2 agonism (**c**) suppress autologous CD8 T cells (*n*=5, *P*>0.05). The difference between standard expansion-mediated suppression (**b**) was statistically different from TNFR2-mediated suppression (**c**), *P*<0.0001.

To functionally characterize the expansion of aTregs in T1D, we investigated their suppressive capacity. We stimulated CFSE-stained autologous T1D PBMC with anti-CD3 mAb and IL-2 in presence of different concentrations of expanded Tregs. The cells were expanded with anti-CD3 plus anti-CD28 plus IL2 (the standard expansion), the standard expansion plus TNF or the standard expansion plus TNFR2 agonism. We measured their suppressive capability by comparing CFSE dilution of CD8 cells within responder PBMC. Our results demonstrated that expanded Tregs from T1D showed dose-dependent suppressive capacity (Figure 6a). We next compared the CD8 suppressive capacity of control and T1D Treg cells expanded with the standard expansion or with TNFR2 agonism (Figure 6b). We wanted to confirm that the expanded Tregs in T1D functioned as potently as controls. The data show the T1D Tregs had suppressive activity comparable to controls under both expansion conditions (Figure 6b). The data also show for both T1D and controls, TNFR2 agonism treatment potentiated the suppressive effect with a change in the slopes using a regression model (Figure 6b versus Figure 6c, *P*<0.0001). Our data suggest T1D patients may have a defect in development of aTregs, but with TNFR2 agonism in culture this Treg maturation defect is corrected. TNFR2 receptor agonism might be a key target molecule for treatment of T1D for *in vitro* or *in vivo* Treg expansion.

## Discussion

Over 25 years ago, T cells from T1D were shown to have an activation defect related to the CD45 protein and its splice variants, CD45RA and CD45RO.^20, 24, 25^ Here we show in T1D a similar activation defect specifically in Tregs, as manifest by a lifelong overabundance of rTregs and an under abundance of aTregs. This Treg activation defect has also been observed in another autoimmune disease, lupus.^5^ We show here that the defect in T1D has clinical significance for T1D, given that lower numbers of aTregs are associated with a trend toward lower C-peptide secretion and poorer glycemic control measured by HbA1c. We also show that this defect can be corrected *in vitro* by TNFR2 antibody agonism. TNFR2 agonism is better than other expansion protocols in expanding Treg cell numbers and promoting the conversion of rTregs into homogeneous aTregs. Finally, we show that the correction of the defect with TNFR2 agonism has functional consequences *in vitro*: it suppresses autologous CD8 T-cell proliferation, which is highly desirable in thwarting autoimmunity.

Tregs have a critical role in regulating the immune response. An under-abundance of Tregs is associated with autoimmunity and an overabundance of Tregs found in cancer and infectious diseases.^7, 8, 9, 10^ A particular type of Treg, the aTreg—which expresses high levels of tumor necrosis factor receptor 2 (TNFR2)—is abundantly found in and around human and murine malignant tumors and in chronic infectious diseases such as tuberculosis where the host's immune response is hampered.^7, 8, 9, 10, 11^ These disease states illustrate the use of TNFR2-expressing Tregs as a very effective strategy to prevent a host immune response even when it is needed in cancer and infections. In both the mouse and human literature, TNFR2-expressing Tregs have been shown to be the most suppressive Tregs identified to date.^6, 9, 13^ Also TNFR2 is required for potent Treg function and disease suppression in animal models of multiple sclerosis such as the EAE mouse model.^36^ aTregs are normally maintained through transmembrane forms of TNF (tmTNF) yet T1D have normal levels of tmTNF so the mechanism behind the paucity of aTregs in T1D is unknown (data not shown).

Treg expansion either *in vitro* or *in vivo* might benefit T1D and other autoimmune patients. TNFR2 is a member of the TNF superfamily that might serve in the future for *in vivo* Treg expansion. Unlike its very similar TNFR1 receptor, which has ubiquitous expression, TNFR2 expression is restricted to the subpopulation of potent Tregs, endothelial cells and neurons making it an attractive target for possible *in vivo* antibody agonistic reagents.^37^ Indeed past toxicology studies have shown in rodent and baboons, no toxicity from *in vivo* agonism of the tissue restricted TNFR2 receptor.^38^ In contrast, TNFR1 agonism *in vivo* induces liver failure, hepatic failure and shock.^39^ To date *in vitro* Treg expansion protocols for autoimmunity and graft versus host disease have advanced to clinic in lieu of direct *in vivo* therapy, as the means for expanding Tregs were too toxic for *in vivo* use. It is possible that the use of the TNFR2 receptor agonism *in vivo* could possibly start to allow autoimmune therapies to achieve *in vivo* Treg expansion methods.^40^ It is also important to note that rapamycin can also promote *in vitro* expansion of functional Treg cells in type 1 patients when added to the standard expansion protocols as published in the past, so all data sets suggest T1D Tregs are responsive to the exogenous signals and can be corrected, at least in culture.^41^

In some forms of human cancer, the expression of TNFR2 on infiltrating Tregs is estimated to be 100 times higher than on circulating Tregs in control subjects,^13^ just the opposite of what is found in autoimmunity. This human data supports the role of Treg overabundance in cancer and under abundance in autoimmunity. In other forms of human cancer, the overall abundance of TNFR2 Tregs is higher than in peripheral blood.^13^ Both murine and human data show that the unique TNFR2 target is preferentially expressed on Tregs and is a functional receptor—indeed, the master switch—for Treg survival.^8^ Tregs either die with TNFR2 blockade or expand with TNFR2 stimulation. TNFR2 stimulation with TNFR2 agonist antibodies has the added advantage of preferentially expanding aTregs and expanding those Tregs into potent TNFR2-expressing cells with high densities of the CD45RO protein. Therefore the TNFR2 surface protein is not merely an identifier of potent Tregs, but is the central switch for Treg maturation, survival and development in adulthood.

In total this work identifies a Treg activation defect in all stages of T1D and correlates the Treg activation defect with a more severe clinical course. The TNFR2 activation receptor for Tregs appears functional in T1D and with agonistic antibodies allows *in vitro* the generation of functional and potent suppressive Tregs. Perhaps future treatments to eliminate autoimmunity could be based on *in vivo* treatment with an immunotherapy composed of TNFR2 agonism.

## Methods

### Human subjects

Blood samples from donors were collected into BD Vacutainer EDTA tubes (BD Diagnostics, Franklin Lakes, NJ, USA). All of the donors provided written informed consent (Protocol #2001P001379) and had T1D of at least 2 years duration. A total of 85 type 1-diabetic human subjects were studied and a total of 60 control subjects were studied in various portions of this study. Baseline characteristics of the subjects used in this study are presented in Supplementary Table 1. Blood was processed within 2 h of phlebotomy. HbA1c measurements were performed by the clinical laboratories at the Massachusetts General Hospital.

### Reagents and flow cytometry

Recombinant human TNF was purchased from Leinco Technologies (St Louis, MO, USA), and recombinant human IL-2 was purchased from Sigma-Aldrich (St Louis, MO, USA). Monoclonal antibodies against TNFR2 were produced in house or purchased from commercial vendors as previously described.^8^ Fluorochrome-conjugated mAbs against human CD4 (RPA-T4), CD25 (M-A251), CD45RA (HI100, 2H2), CD45RO (UCHL1, 4HB), CD127 (hIL-7R-M21), HLA-DR (L243 (G46-6)) were purchased from BD Biosciences. Fluorochrome-conjugated monoclonal antibodies against CD4 (S3.5, Invitrogen, Carlsbad, CA, USA), CD120a (16803 R&D systems), CD120b (22235, R&D systems, Minneapolis, MN, USA), FOXP3 (259D, Biolegend, San Diego, CA, USA) were also used in this study.

Intracellular staining of FOXP3 and CD152 was performed using either FOXP3 Fix/Perm Buffer set (Biolegend) or Human FoxP3 Buffer set (BD Biosciences) according to the manufacturer's instructions. Flow cytometric data were obtained using FACSCalibur (BD Biosciences) flow cytometer. All the data were analyzed with Cellquest Software (BD Biosciences, San Jose, CA, USA).

### CD4 cell isolation and induction of aTregs

CD4 T cells were isolated from fresh human blood within 2 h of venipuncture using Dynal CD4 Positive Isolation Kit (Invitrogen). We modified the protocol recommended by the manufacturer by using Hanks' Balanced Salt Solution supplemented with 2% fetal bovine serum (FBS) (Hyclone, Logan, UT, USA) instead of PBS. The quality of isolated cells was assessed to be >98% in purity and 96% in viability by CD4 and propidium iodide staining.

For induction of the aTreg experiment, 2 × 10^5^ cells of freshly isolated CD4 cells were plated in 96 round-bottom well and treated with IL-2 (50 U ml^−1^) and TNF (20 ng ml^−1^) or TNFR2 mAb (2.5 μg ml^−1^). After 16-48 h, cells were collected and were determined by flow cytometry.

### Isolation and expansion of CD4^+^CD25^+^ cells

Extraction of CD25 positive cells was subsequently performed after CD4 isolation using Dynabeads CD25 and DETACHaBEAD CD4/CD8 (Invitrogen). After isolation, 2 × 10^4^ cells were cultured in 96 round-bottom well plate in culture medium (RPMI 1640 medium supplemented with 10% FBS, 2 mm Glutamax, 100 U ml^−1^ penicillin and 100 μg ml^−1^ streptomycin (Invitrogen). Dynabeads Human T_reg_ Expander (Invitrogen) was added at a beads-to-cell ratio of 2:1. This was called a standard method. The expander was a mixture of anti-CD3 and anti-CD28 antibodies plus IL2. Also some expansion protocols also had added rapamycin (1 μM, EMD Biosciences, San Diego, CA, USA) plus TNF (20 ng ml^−1^), or plus TNFR2 agonistic antibody (2.5 μg ml^−1^, Immunobiology Core, MGH, Boston, MA, USA). After two days, IL-2 (200 U ml^−1^) was added to the culture. Half of the media was changed every 2–3 days containing rapamycin (until day 7) and 100 U ml^−1^ of IL-2. On day 9, additional TNF or TNFR2 mAbs were supplied into the media. On day 16, cells were collected, Dynabeads Human T_reg_ Expander was removed, washed and rested at 37 °C in a humidified 5% CO_2_ incubator in RPMI 1640 medium supplemented with 1% FBS, 2 mm Glutamax, 100 U ml^−1^ penicillin, 100 μg ml^−1^ streptomycin and 10 U ml^−1^ IL-2. On the following day, cells were counted using hemacytometer, and their phenotype was analyzed by flow cytometer.

### Intracellular cytokine staining

Expanded CD4^+^CD25^+^ cells were stimulated with phorbol myristate acetate (2 ng ml^−1^) and ionomycin (500 ng ml^−1^) (Sigma) for 24 h. Monensin (GolgiStop, BD Biosciences) was added for the last 4 h of incubation. Cells were fixed and permeabilized using Human FOXP3 Buffer Set, followed by staining with fluorochrome-conjugated IL-10 (JES3-9D7) and IL-17A (N49-653-19F1, BD Biosciences) mAbs.

### Cell proliferation and suppression assays

For PBL proliferation experiments, PBLs were stained with 1 μm carboxyfluorescein diacetate succinimidyl ester (CFSE). Cells were plated at the density of 2 × 10^5^ cells per well in 96-well plate precoated with anti-CD3 mAbs (5 μg ml^−1^) (OKT3, eBiosciences, San Diego, CA, USA). Four days later, cells were collected and analyzed by flow cytometry. The proliferation rate was calculated by the percentage of cells undergoing division.

For T_reg_ suppression assay, autologous PBMCs were used as responder cells. PBMC was collected at the day of venipuncture by density gradient separation using Ficoll-Paque Plus (GE Healthcare, Piscataway, NJ, USA) cryopreserved at −80 °C, and thawed at the day before mixed with T_regs_ and rested overnight in RPMI 1640 medium supplemented with 1% FBS, and 10 U ml^−1^ IL-2. On the following day, responder cells were stained with CFSE (1 μm). Responder cells (5 × 10^4^ cells) and expanded T_regs_ were mixed at the ratio of 1:1, 2:1 and 4:1 in culture media, and stimulated with anti-CD3 mAb (HIT3a, BD Biosciences) and IL-2 (50 U ml^−1^). After 4 days, cells were collected and analyzed by flow cytometry. Suppression index was calculated by the percentage of CD8 cells in responder cells that underwent division. Suppression index was calculated using following equation: (T_Resp_ proliferation without T_Reg_−T_Resp_ proliferation with T_Reg_)/T_Resp_ proliferation without T_Reg_.

### C-peptide assay methods

Serum samples were assayed for C-peptide with the Mercodia AB (Uppsala, Sweden) regular (Cat. No 10-1136-01) or ultrasensitive C-peptide ELISA kits (Cat. No 10-1141-01). Both assays were calibrated against the International Reference Reagent for C-peptide, IRR C-peptide 84/510 (a WHO standard) and listed with FDA as Class I IVD devices. The lower limit of sensitivity of the Mercodia ultrasensitive assay for these studies was calibrated to 5.0 pmol l^−1^. Additional details have been previously reported.^34^

### Statistical analysis

Data analyses were performed by the paired Student's *t-*test, Pearson's correlation coefficient and ANCOVA using GraphPad Prism 5 software (GraphPad Software, La Jolla, CA, USA). We considered two-sided *P*-value 0.05 as significant without controlling for multiple comparisons. For the slopes of correlations *R* values were calculated by Spearman correlation and *P*-values calculation by ANCOVA.
