# Supplementary material for: Treg activation defect in type 1 diabetes: correction with TNFR2 agonism
Source: Clin Transl Immunology. 2016 Jan 8;5(1):e56–. doi: 10.1038/cti.2015.43 (PMC4735064; doi:10.1038/cti.2015.43)
Supplement: Supplementary Table 1 [file cti201543x1.pdf]

	Type 1 Diabetes (N = 92)	Control (N= 61)	
Age (yrs)	36.9 ± 16.6 (6 - 67)	39.5 ± 14.0 (17 - 68)	P = 0.313
Gender (M / F)	55 / 37	32 / 29	NA
Age of onset (yr)	18.0 ± 14.1 (2 - 57)	NA	NA
Duration (yr)	18.9 ± 13.5 (1 - 54)	NA	NA
HbA1c (%)	7.61 ± 1.13 (5 - 12)	NA	NA
