# Supplementary figures and images for: Treg activation defect in type 1 diabetes: correction with TNFR2 agonism
Source: Clin Transl Immunology. 2016 Jan 8;5(1):e56–. doi: 10.1038/cti.2015.43 (PMC4735064; doi:10.1038/cti.2015.43)

# Supplemental Figure 1

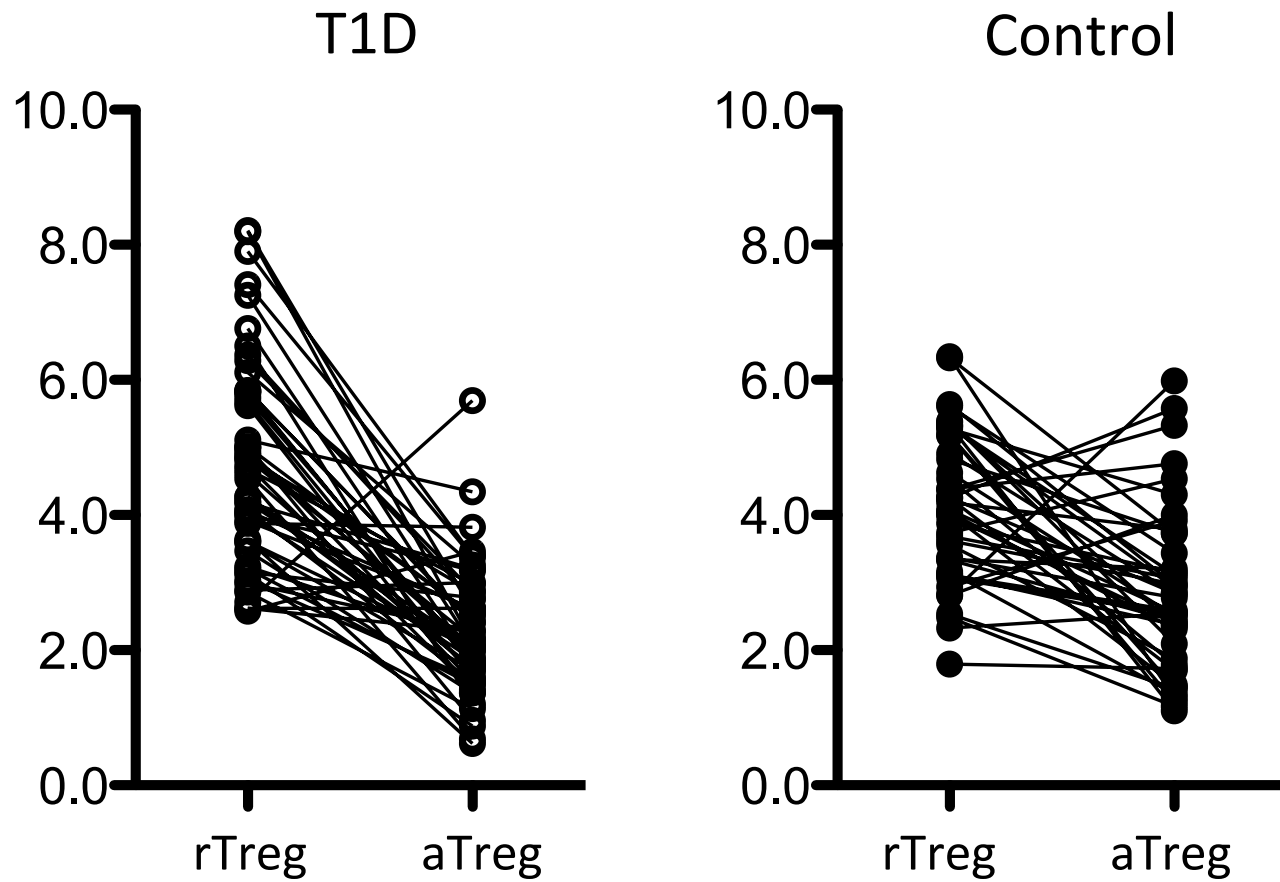

Supplement: Supplementary Figure 1 [file cti201543x2.pdf]

a Standard

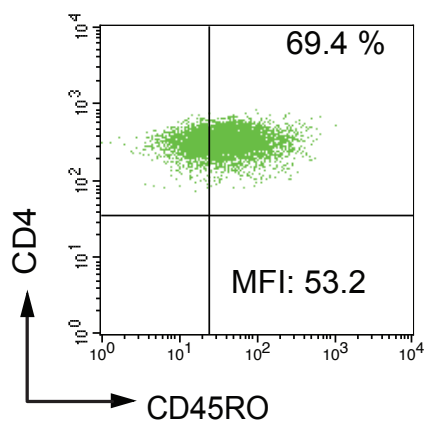

b TNF

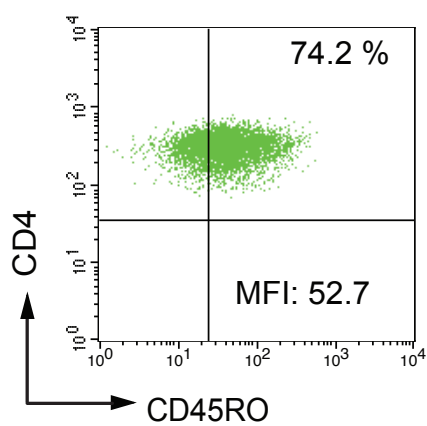

c TNFR2 agonist

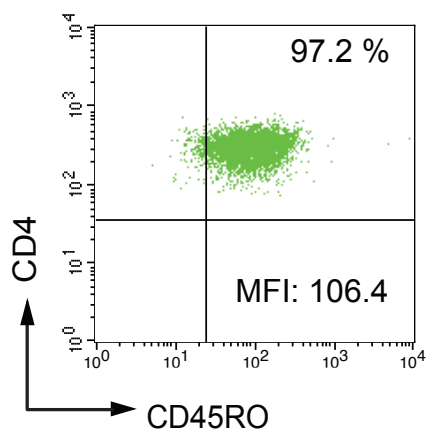

Supplement: Supplementary Figure 2 [file cti201543x3.pdf]
