# Supplementary material for: Treg activation defect in type 1 diabetes: correction with TNFR2 agonism
Source: Clin Transl Immunology. 2016 Jan 8;5(1):e56–. doi: 10.1038/cti.2015.43 (PMC4735064; doi:10.1038/cti.2015.43)
Supplement: Supplementary Legends [file cti201543x4.doc]

**Supplementary Information:**

**Supplementary Table 1**: T1D and Control Subject Traits for the data in Figures 1-6.

**Supplementary Figure 1:** Paired characterization of activated and resting Tregs from T1D subjects (n=55) compared to control subjects (n=45). Type 1 diabetics have fewer aTregs (p=0.01) and more rTregs (p=0.02).

**Supplementary Figure 2**: Illustrative histograms of expanded Tregs during the 17 days of co-culture with anti-CD3 and anti-CD28. Cells expanded with TNFR2 agonism addition can had higher numbers of CD45RO+ cells (97.2%) compared to the standard expansion group (69.4%) or the TNF treatment group (74.2%), as measured by cell counts. The TNFR2 agonism expanded Tregs also always had higher mean fluorescence intensity (MFI) of the CD45RO protein (106.4 MFI) compared to standard expansion (MFI 53.2) or to TNF expansion (MFI 52.7).
